# Reactive perforating collagenosis successfully treated with dupilumab

**DOI:** 10.1111/ajd.13874

**Published:** 2022-05-28

**Authors:** Javier Gil‐Lianes, Constanza Riquelme‐Mc Loughlin, Jose Manuel Mascaró

**Affiliations:** ^1^ Dermatology Department Hospital Clínic de Barcelona Universitat de Barcelona Barcelona Spain

Dear Editor,

A woman in her 40s with a history of atopic dermatitis in her childhood and no other medical history consulted with a disseminated, pruritic, papulonodular eruption of 7 months of duration. On examination, the patient had multiple papules and nodules with a crateriform appearance, an erythematous border and a large central necrotic crust, affecting the trunk, arms and legs (Figure 1a–c). There was no palmar‐plantar nor mucosal involvement.

**FIGURE 1:**
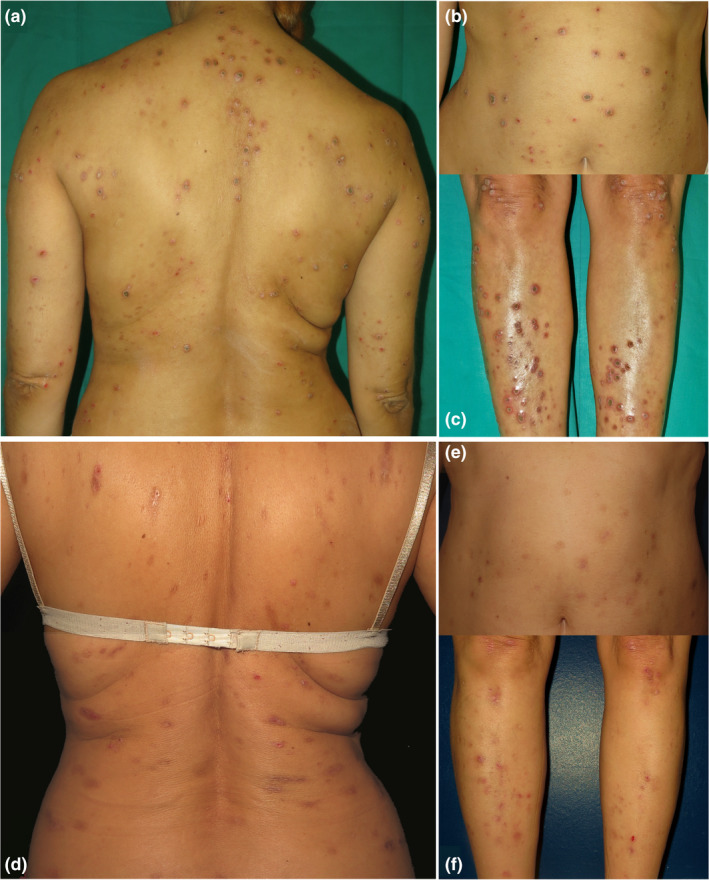
Acquired perforating dermatosis. Baseline and after dupilumab treatment. a‐c. Multiple umbilicated papules and nodules with a central keratotic crust and erythematous margins, affecting the trunk, arms and legs. d‐f. Residual hyperpigmented lesions, atrophic scars and isolated superficial erosions, without active nodular lesions

The histopathological study showed epidermal invagination with signs of ulceration and transepidermal elimination of collagen, accompanied by a marked intraepidermal inflammation with polymorphonuclear leukocytes without eosinophils. In the dermis, there was a mixed inflammatory infiltrate composed of polymorphonuclear and histiocytic cells.

Complete blood count, chemistry profile, and hepatic, renal and thyroid function were normal except for eosinophilia (700 × 10^6^/L), elevated non‐specific IgE (519 kU/L) and positive antinuclear antibodies (320 URF) without any antigenic specificity.

A diagnosis of reactive perforating collagenosis was made. Treatment with topical corticosteroids and narrowband (NB) UVB, and later psoralen plus ultraviolet A (PUVA), was started with a partial response after 8 months. Subsequently, the patient received different treatments: oral corticosteroids, antihistamines, topical corticosteroids under occlusion and cyclosporine with partial improvement and repeated flares. Three years after diagnosis, subcutaneous dupilumab was prescribed with a loading dose of 600 mg and then 300 mg fortnightly, together with NB‐UVB for 6 weeks. After 2 months, there was improvement in pruritus without new lesions. After 12 months, the patient presented a nearly complete response, with only residual hyperpigmentation and marked improvement in her quality of life (Figure 1d–f) which persists after 24 months with dupilumab therapy.

Acquired perforating dermatoses (APD) are a rare group of skin disorders of unknown aetiology characterised by transepidermal elimination of dermal material of which reactive perforating collagenosis is the most common. Although its pathogenesis remains unclear, pruritus and repeated trauma from scratching are regarded as central pathogenic factors.^1^

Treatment for APD can be challenging. The most common treatments are topical and intralesional steroids, oral antihistamines and topical retinoids. Other treatment options include NB‐UVB, PUVA, oral retinoids, allopurinol, tetracyclines, dapsone, hydroxychloroquine, methotrexate and apremilast with variable results.^1^ Recently, Kawakami et al. published the first clinical practice guide for the treatment of perforating dermatosis^2^ where they follow a multimodal approach, focusing on the underlying diseases and pruritus.^1^, ^2^ This can include metabolic control of diabetes, dialysis of CKD or treatment of existing neoplasms.^1^, ^2^

Chronic pruritus found in APD mostly depends on a non‐histaminergic pathway^3^ with interleukin (IL)‐4, IL‐13 and IL‐31 acting as regulators of chronic itch. Dupilumab, a recombinant human monoclonal antibody directed against the IL‐4 receptor‐α subunit of IL‐4 and IL‐13 receptors, has demonstrated antipruritic properties in atopic dermatitis and PN.^3^ Additionally, previous studies have shown that dupilumab effectiveness in PN is independent of a past medical history of atopy.^4^ Ying et al recently reported two patients with a perforating collagenosis and elderly atopic dermatitis who presented a complete response after 3 months with dupilumab.^5^

Treatment of APD is directed towards aetiological factors and pruritus control, with usually poor response to most therapies. Given the role of IL‐4 and IL‐13 in chronic pruritus, dupilumab could represent a potential treatment for patients with APD. Future research should be directed towards targeted therapies such as anti‐IL 4, 13 and 31 or JAK inhibitors in APD.

## FUNDING INFORMATION

The authors involved have reported no relevant financial relationships with commercial interest(s).

## CONFLICT OF INTEREST

Dr. Riquelme has received honoraria as a consultant for Lilly and has received speaker fees from Sanofi, both outside the submitted work. Dr. Mascaró has received speaker fees from Academia Española de Dermatología, Bocemtium Consulting, Clover Soluciones Globales de Marketing, Ferrer Internacional, Fundació Clínic per la Recerca Biomédica, LEO Pharma Spain, Loki & Dimas, Luzan 5 Health Consulting, and M.S.D. de España S.A, S&H Medical Science Service, and Sanofi‐Aventis all outside the submitted work.

## INFORMED CONSENT

The patients in this manuscript have given written informed consent to the publication of their case details.
